# Unusual presentation of spontaneous pneumomediastinum

**DOI:** 10.4103/0970-2113.71961

**Published:** 2010

**Authors:** Tae jin Cho, Hoon Kim

**Affiliations:** *Department of Emergency Medicine, College of Medicine, Inha Unversity Hospital, Incheon, Korea*; 1 *Department of Emergency Medicine, Inje University Ilsan Paik Hospital, Goyang, Korea*

**Keywords:** Dysphagia, pneumomediastinum, spontaneous

## Abstract

Spontaneous pneumomediastinum (SPM) is an uncommon, self-limiting condition resulting from alveolar rupture in young adults. There are asthma, illicit drug use, and activities triggering a Valsalva maneuver as causes of developing SPM. We report two patients who were diagnosed with SPM in the absence of known predisposing factors and without any clinical sign of subcutaneous emphysema of the neck, the most common physical finding on presentation. Both of them developed dysphagia after swallowing a peach seed and boned rib of pork, respectively. SPM was suspected after performing lateral neck X-ray, and the diagnosis of SPM was confirmed by chest CT. These cases showed the importance of performing the lateral neck X-ray to screen SPM in patients with dysphagia.

## INTRODUCTION

Spontaneous pneumomediastinum (SPM) is a rare, generally benign condition, characterized by the presence of air in the mediastinum without obvious preceding cause. Its incidence has been reported as approximately 1 in 30,000 emergency department (ED) referrals.[1] Known risk factors are asthma, illicit drug use, smoking, activities triggering Valsalva maneuver. We report two cases of SPM complaining of dysphagia after dinner without any predisposing factors.

## CASE REPORTS

### Case 1

A 16-year-old man presented to the ED with the sensation of a foreign object in his throat 30 minutes after swallowing a peach seed by mistake at dinner. He complained of odynophagia and neck discomfort. He denied neck trauma or any activity that may result in a Valsalva maneuver such as coughing, retching or vomiting; and the use of any medications or illicit drug. He did not have remarkable medical history, especially any pulmonary diseases, as asthma. He was a non-smoker and healthy high-school student.

On physical examination, his blood pressure was 110/80mmHg, with pulse rate 82 beats/min. respiratory rate 18 breaths/min, and body temperature of 36.0’C. His height was 175 cm, weight 80 kg. There was no crepitus on neck. There was no foreign body or abnormal finding in the oropharynx. Laryngopharygoscopy did not reveal mucosal lesions or signs of submucosal swelling, and did not reveal any foreign body in the airway above the vocal cords. The heart sounds were normal. The trachea was in central position, and chest percussion was and breath sounds were all normal.

**Figure 1:**
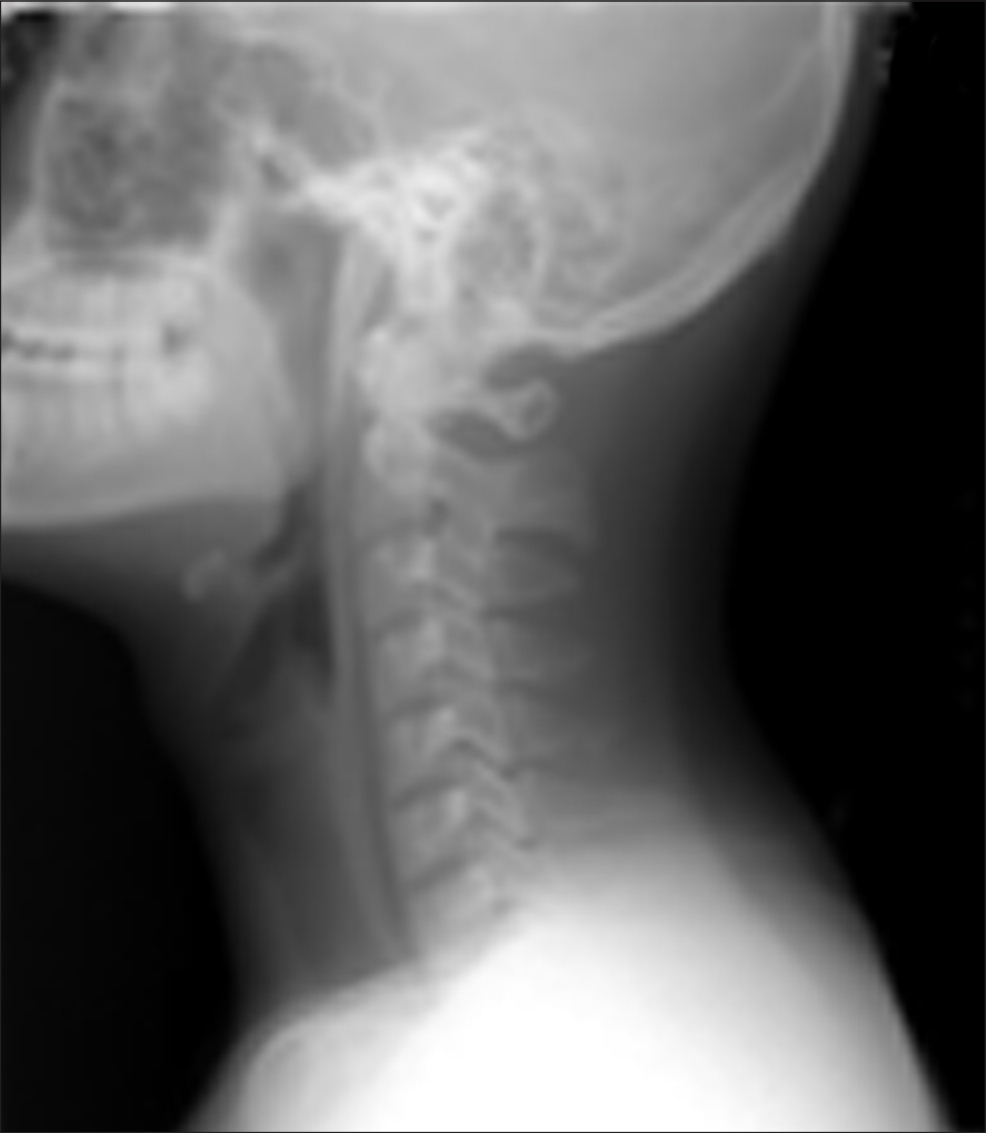
Lateral neck X-ray case 1 reveals emphysema in the prevertebral space

**Figure 2:**
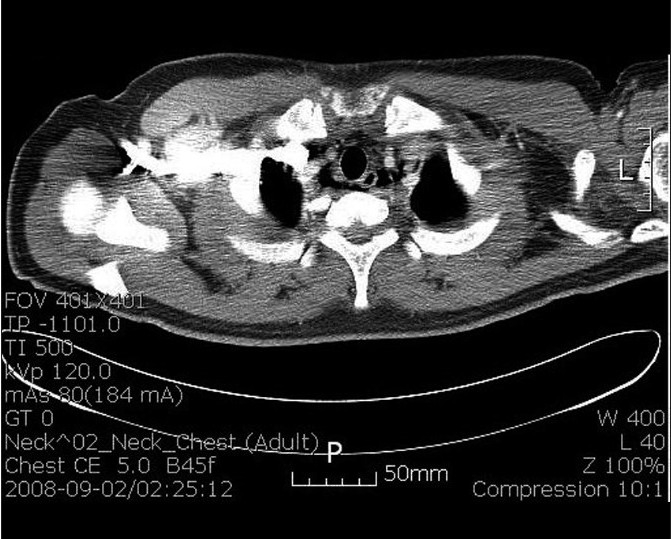
Chest CT scan of case 1 reveals pneumomediastinum

The patient had a normal ECG. Chest X-ray did not show subcutaneous emphysema and active lesions in the lungs. Blood tests revealed no abnormalities. However, lateral neck X-ray revealed emphysema in prevertebral space, C-spine [Figure 1]. A subsquent CT of the neck and chest revealed emphysema of both neck retropharyngeal area with pneumomediastinum [Figure 2]. There was no evidence of foreign body or pneumothorax or esophageal rupture. Esophagogram demonstrated a good passage of barium in esophagus without obstruction or leakage.

The patient was admitted and treated conservatively. The patient’s progress was uneventful and after two days he was discharged.

### Case 2

A 19-year-old woman presented to the emergency department with odynophagia, neck discomfort, dypnea and chest discomfort. The onset of her symptoms was while sleeping in the bus 45 minutes after eating boned rib of pork as dinner. She denied recent upper respiratory infection, neck or chest trauma and any activity such as coughing, sneezing. She denied vomiting, and the use of any medications or illicit drug. She did not have remarkable medical history, especially asthma except appendectomy seven years ago. She was a non-smoker and a healthy university student.

On physical examination, her blood pressure was 130/90mmHg, pulse rate 72 beats/min, and the respiratory rate was 20 breaths/min with oxygen saturation of 100% on room air. Body temperature was 36.0’C. Her height was 162 cm, and the body weight was 49 kg. There was no crepitus on neck and, there was not foreign body or abnormal finding in the visual oropharynx exam. The heart sounds were normal. The trachea was in central position, and chest percussion and breath sounds were all normal.

**Figure 3:**
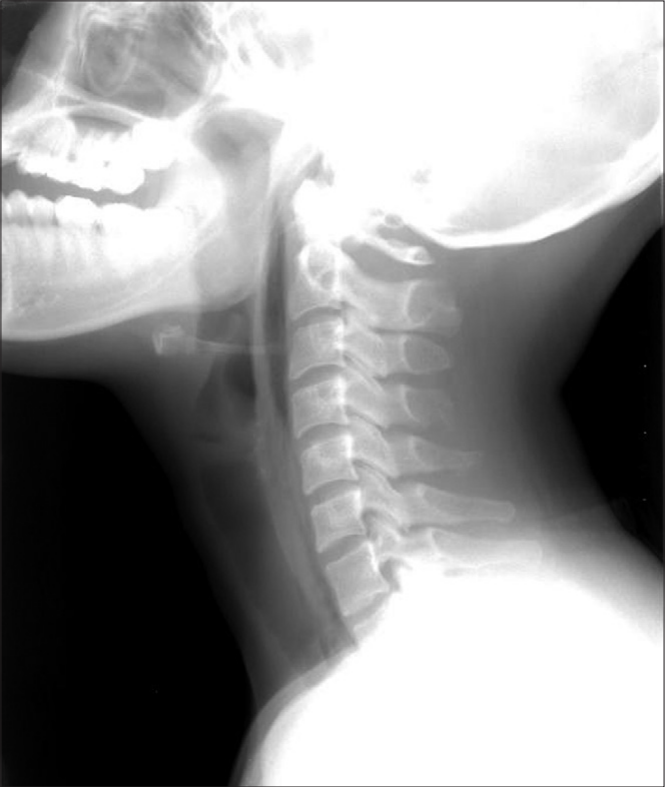
Lateral neck X-ray of case 2 reveals emphysema in the prevertebral space

**Figure 4:**
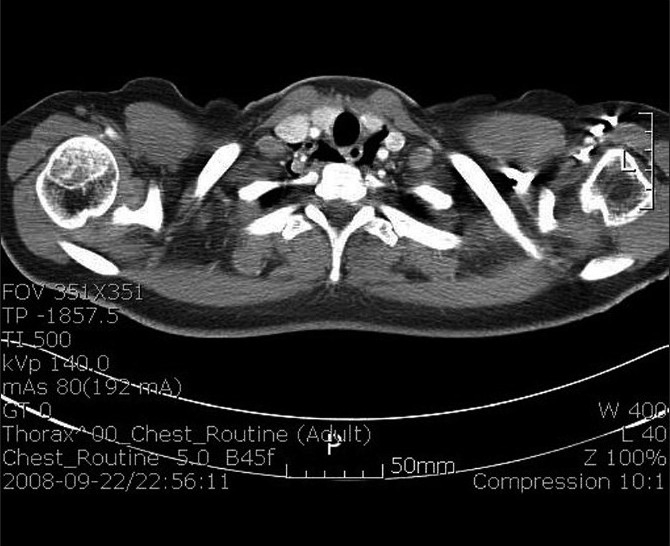
Chest CT scan of case 2 reveals pneumomediastinu

Routine laboratory tests, cardiac enzymes and an electrocardiogram were normal. Chest X-ray did not show subcutaneous emphysema and active lesions in the lungs. However lateral neck X-ray revealed emphysema in prevertebral space, C-spine [Figure 3]. A subsquent CT scan of the chest revealed pneumomediastinum and neck soft tissue emphysema [Figure 4]. There was no evidence of foreign body or pneumothorax or esophageal rupture. Esophagogram demonstrated a good passage of barium in esophagus without obstruction or leakage.

Dysphagia disappeared without specific treatment, and the patient was discharged four days after admission without further medical evaluation.

## DISCUSSION

First report of cases of SPM was in 1939 by Hamman.[2] A number of cases have been reported with various conditions. Secondary pneumomediastinum should be excluded before diagnosing SPM. Secondary pneumomediastinum have specific responsible pathologic event, such as blunt or penetrating trauma, pulmonary or mediastinal infection by gas-producing organism, and esophageal rupture after vomiting.[3]

Pathophysiology of SPM is suggested as alveolar rupture following an acute rise in intra-alveolar pressure. The air ascends along the mediastinum toward the subcutaneous space of the neck, causing cervicofacial subcutaneous emphysema.[4]

Asthma, illicit drug use, childbirth, forceful straining, coughing, sneezing, retching or vomiting, all activities that require the Valsalva maneuver and intense breathing work or exercise are well-known predisposing factors of SPM.[5] In addition, malnutrition associated with anorexia nervosa and self-induced oral injury are reported as predisposing factor of SPM.[6–7]

The most common symptoms include acute central chest pain, which can radiate anteriorly, posteriorly and to the jaw, dyspnea, neck pain and swelling and hoarse voice. Dysphagia, cough, odynophagia and dysphonia are much less frequent findings. The reported prevalence of subcutaneous emphysema on the neck is ranging from 40% to 100%.[1,3,5] Hamman’s sign is a distinctive crunching or bubbling sound that is synchronous with the heartbeat, and recently reported prevalence was 12%.[5]

The diagnosis of SPM is dependent on radiologic imaging. In a percentage up to 30% of patients with SPM the chest radiograph is normal. So, CT scan of the chest is considered the gold standard for diagnosing pneumomediastinum.[8] Complementary diagnostic procedures such as esophagogram, esophagoscopy, and bronchoscopy may be needed to rule out spontaneous (Boerhaave syndrome) or traumatic rupture of esophagus and tracheobrochial tree.

Our two patients mainly complained of odynophagia and, did not have any clinical sign of subcutaneous emphysema of the neck, Hamman’s sign and known predisposing factors such as asthma. Because symptoms were developed after eating foods, foreign material in the throat rather than SPM was suspected initially. Accidental swallowing of a dump of food could have caused an increased intrathoracic pressure and SPM in our cases. Although it is difficult to draw any firm conclusion based on two cases, we suggest that lateral neck X-ray should be considered as more appropriate screening test for disclose an SPM than chest X-ray in patients with these clinical situations.
